# Novel Mutations in Pilomatrixoma, CTNNB1 p.s45F, and FGFR2 p.s252L: A Report of Three Cases Diagnosed by Fine-Needle Aspiration Biopsy, with Review of the Literature

**DOI:** 10.1155/2020/8831006

**Published:** 2020-08-29

**Authors:** Cristina Aparecida Troques da Silveira Mitteldorf, Rafael Sarlo Vilela, Melissa Lissae Fugimori, Carla Daniele de Godoy, Renata de Almeida Coudry

**Affiliations:** ^1^Laboratory of Anatomic Pathology, Hospital Sírio Libanês, Rua Dona Adma Jafet 91, 6° Floor, Block E, São Paulo, SP 01308-050, Brazil; ^2^United Health Group Brazil, Rua Enxovia, 461–467, Chácara Santo Antônio, São Paulo, SP 04711-050, Brazil

## Abstract

Pilomatrixoma (*calcifying epithelioma of Malherbe*) is an uncommon benign skin appendageal tumor that differentiates toward hair matrix cells. It is misdiagnosed in up to 75% of cases by nondermatologists. Although the histopathological findings are well recognized and characteristic, diagnosis by fine-needle aspiration biopsy may be quite challenging. Several reports have emphasized the challenges in cytodiagnosis of pilomatrixoma, leading to a false-positive diagnosis. The lesions may show avidity for fludeoxyglucose on positron emission tomography/computed tomography scan, raising concern of a possible malignant neoplasm. CTNNB1 mutations have been reported in a high percentage of pilomatrixomas. Expression of *β*-catenin, the protein encoded by CTNNB1, is also frequently observed. To determine if routine cytological specimens can be successfully used to perform additional investigation and support or confirm the diagnosis in three cases of pilomatrixoma, we performed molecular analysis and immunohistochemistry to search for CTNNB1 mutation and *β*-catenin, respectively. *β*-Catenin positivity by immunohistochemistry was observed in basaloid cells in all three cases. Exon 3 mutations in *CTNNB1* were detected in all cases. In addition, we detected a fibroblast growth factor receptor 2 (FGFR2) mutation in one of the cases. We reviewed the literature and present the clinical and morphological characteristics that must be considered along with other findings to accurately achieve the correct diagnosis, in correlation with the results of the ancillary technique. In conclusion, routine cytological specimens can be successfully used to perform additional investigations and support cytodiagnosis in difficult cases.

## 1. Introduction

Pilomatrixoma, also known as *calcifying epithelioma of Malherbe*, is a benign skin appendageal tumor that differentiates toward hair matrix cells. The exact prevalence is unknown, but pilomatrixoma accounts for approximately less than 1% of all benign skin tumors. It presents most frequently as a firm to hard intradermal/subcutaneous small tumor, in the head and neck, during the first two decades of life, with a slight predominance in females, and is misdiagnosed in up to 75% cases by nondermatologists [1–3].

The lesion may show avidity for fludeoxyglucose (FDG) on positron emission tomography/computed tomography (PET/CT) scan, raising concerns of a possible malignant neoplasm [4–6].

The histopathological findings are well recognized and characteristic, but diagnosis by fine-needle aspiration biopsy (FNAB) may be quite challenging. In a retrospective analysis, Woyke et al. first described the cytological features of pilomatrixoma in six cases, four of which were misdiagnosed as malignant tumors [7].

Since then, several reports, mostly short series, and isolated cases have emphasized the difficulty in cytodiagnosis of pilomatrixoma, which may lead to a false-positive diagnosis [8–48].

CTNNB1 mutation seems to be present in almost all cases of pilomatrixomas [49]. The protein encoded by CTNNB1, *β*-catenin, is also frequently detected by immunostaining, but conflicting results have been reported [50–55].

We present three cases of pilomatrixoma diagnosed by FNAB in which we were able to confirm CTNNB1 mutations using routine cytological specimens. The *β*-catenin protein expression was also detected in cell block preparations by immunohistochemistry. Besides reporting cytomorphological findings and reviewing the literature, our aim was to confirm that routine cytological specimens can be successfully used to perform additional investigations and support or confirm the diagnosis in difficult cases.

## 2. Case Presentation

All three cases involved women, aged 6, 13, and 38 years, presenting discrete painless nodules in the preauricular region, measuring 1.0, 1.4, and 1.4 cm, respectively; through clinical evaluation, these were initially interpreted to be of parotid gland origin.

FNAB was performed using 23–25 gauge needles and conventional techniques. Ultrasound-guided punctures were performed through three passes per nodule in all cases. We systematically used different cytological preparations from the aspirated samples, including conventional smears and liquid-based and cell block specimens [56].

Before the final diagnosis, correlation with ultrasound findings was helpful, as nodules in all cases seemed to be superficially located, outside the parotid gland, possibly in the skin, with a hyperechoic component, which is suggestive of calcification. In fact, the presence of calcification could even be inferred by lesion consistency during the procedure.

**Figure 1:**
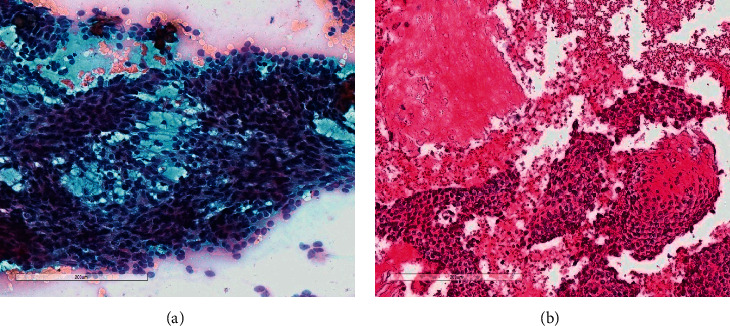
(a) Conventional smear showing basaloid cells in sheets, with focal squamous appearance (Papanicoloau stain, 100x) and (b) cell block preparations confirming the presence of basaloid cells with squamous differentiation, in addition to ghost cells (H&E, 100x).

The specimens showed adequate cellularity in all cases, and many cytomorphological aspects of the lesion could be appreciated in one or more of the preparations used. Figures 1(a) and 1(b) illustrate the morphological findings of the routine specimens.

The most important and constant finding was the presence of small basaloid epithelial cells, predominantly clustered, without peculiar arrangement, with slight size variation, but without significant atypia. Focal transition to elongated and keratinized cells was observed. Numerous cell elements without nuclei and amorphous material, consistent with “shadow cells,” were present in different amounts, with calcium deposits. Occasional, isolated, multinucleated giant cells and mild inflammatory components were also noted. No necrosis or significant mitotic activity was observed.

Diagnosis was signed out as benign, basaloid epithelial neoplasia, with a note recommending close clinical correlation, suggesting the possibility of cutaneous/dermal lesions, originating from appendages, mostly pilomatrixoma.

Two cases were surgically resected, and the diagnosis of pilomatrixoma was confirmed. The surgeon and the family decided to follow conservatively but observe closely the nodule from the youngest patient.

### 2.1. Immunohistochemistry

Cell block specimens were used to test the expression of *β*-catenin by immunohistochemistry. Formalin-fixed and paraffin-embedded cell blocks were sectioned, and 4 *μ*m sections were processed on the Ventana Benchmark ULTRA automated stainer using a prediluted monoclonal mouse anti-human *β*-catenin antibody (clone 14), ultraView Universal DAB detection kit, and hematoxylin II counterstain, as recommended (Roche Ventana, Tucson, AZ). Positive and negative controls were included. Immunohistochemistry was positive in basaloid cells from all cases, with different expression patterns; one case showed exclusively membranous staining, and in the other two cases, cytoplasmic staining, with equivocal, weak nuclear positivity, was observed. The transitional cells did not show positivity in any case.

Data from the described cases are summarized in Table 1.

### 2.2. Molecular Analysis

Targeting amplicon panel contains 26 genes related to solid tumors. Cytological smears have already been established at the Molecular Pathology Laboratory of Hospital Sírio-Libanês (publication in progress). All genes present in the panel are listed in Supplementary Table 1.

One conventional smear containing 3,000–15,000 cells and a range of tumor cells between 60% and 80% was selected for DNA isolation from each case.

The coverslip was removed with xylene, and then the slide was washed with xylene and 100% ethanol to remove any residual mounting medium. The entire smear was scraped off the slide with a razor blade and placed on a microtube to be digested with protease for 3 h at 56°C. DNA isolation was performed with QIAamp DNA Micro Kit (Qiagen), and DNA concentration was determined using a Qubit 2.0 fluorimeter (Life Technologies).

DNA quality was assessed by amplifying an endogenous gene by the real-time PCR assay, which is included in the library construction kit (Trusight Tumor 26 kit, Illumina). ΔCt <6 was considered acceptable to proceed with testing.

Library construction was performed according to the manufacturer's instructions, and the libraries were sequenced at MiSeq (Illumina) using the v2 300 cycles cartridge. Data were analyzed using the MiSeq reporter for alignment to the human genome hg19, and the variants were identified on the Amplicon DS workflow provided by Illumina. The average read depth was higher than 20,000x for all samples.

Exon 3 *CTNNB1*mutations were detected in all three cases. The two cases with cytoplasmic and equivocal nuclear staining presented the same *CTNNB1 p.*S33Fmutation (cases 1 and 2). In the case with a membranous staining pattern, *CTNNB1*p.S45F mutation was detected (Case 3). In Case 1, we also detected an *FGFR 2*p.S252L mutation.

## 3. Discussion

We were able to detect exon 3 *CTNNB1* mutations in all three cases of pilomatrixoma, using routine cytological specimens obtained by FNAB.

*CTNNB1* encodes the *β*-catenin protein, which seems to possess a multifaceted nuclear function that may significantly impact the initiation and progression of numerous human tumors.

Somatic mutations in *CTNNB1,* which affect the N-terminal segment of *β*-catenin, are found in at least 75% of human pilomatrixomas, leading to a constitutively overactive encoded protein. Transgenic mice expressing activated *β*-catenin are predisposed to the development of skin tumors resembling pilomatrixomas [49]. This percentage of mutations was greater than in all other human tumors examined, suggesting that the dysregulation of *β*-catenin is a major cause of pilomatrixomas in humans.

Nearly all *CTNNB1* mutations reported in human cancers are localized in exon 3, and most of them occur at serine-threonine sites or on adjacent residues.

p.S33F mutation was detected in two cases, and p.S45F mutation was detected in another case. Both *CTNNB1* mutations, p.S33F and p.S45F, are within the ubiquitination recognition motif of the *CTNNB1* protein, at the Gsk3b phosphorylation site of *β*-catenin. S33F is predicted to confer a gain-of-function to the protein, as demonstrated by its nuclear accumulation [57–59]. S45F also confers a gain-of-function to *β*-catenin and increases CTNNB1-dependent transcription [60].

As far as we are concerned, *CTNNB1* p.S45F mutation has not been reported previously in pilomatrixoma but was described in neoplasms of other sites, such as the colorectum, endometrium, stomach, liver, and central nervous system [61].

Because our cases were submitted to a panel of genetic markers, we also detected an *FGFR2* p.S252L mutation in one case, which is a novel gene alteration that has not been reported previously in pilomatrixoma. FGFR comprises a family of related, but individually distinct, tyrosine kinase receptors. *FGFR2* mutations have been reported in tumor specimens originating from the prostate, breast, lung, uterine, and ovarian tumors [62].

FGFR2 p.S252L lies within the extracellular domain of FGFR2 (UniProt.org) and has not been functionally or clinically validated but is considered to be likely oncogenic although its effect on FGFR2 protein function is unknown.

*β*-Catenin is an effector of intercellular adhesion and participates in the Wnt signaling pathway. Normally, *β*-catenin that is not assembled into adherent junctions becomes phosphorylated at its N-terminal segment, thereby targeting the protein for ubiquitin-mediated degradation. Elevation of the cytoplasmic pool of *β*-catenin occurs by the binding of Wnt ligands or by gene mutations in CTNNB1, APC, or AXIN. Accumulation of cytoplasmic *β*-catenin results in its binding to members of the lymphoid enhancer factor (LEF) family and the entry of these binding proteins into the nucleus. In the nucleus, *β*-catenin functions both as an activator and repressor of transcription in a context-dependent manner. It promotes cell proliferation and supports tumor growth by enhancing angiogenesis. *β*-Catenin-mediated signaling regulates cancer cell metabolism and has been associated with tumor-initiating cells in multiple malignancies. In addition, it functions both as a pro- and antiapoptotic factor, while also inhibiting the recruitment of inflammatory antitumor T cells [63].

Consequently, cellular *β*-catenin exists in three different pools: membranous, cytoplasmic, and nuclear. This has been demonstrated by immunohistochemistry of component cells in pilomatrixoma, presenting with membranous, cytoplasmic, and nuclear patterns, as expected.

Besides the CTNNB1 mutation, we observed *β*-catenin immunoexpression in all three cases. The pattern of *β*-catenin expression in this type of lesion (nucleus, membrane, or cytoplasm) and the cellular type (basaloid, transitional, or shadow cells) varies among different studies [50–55]. Furthermore, the relationship between cellular *β*-catenin localization detected by immunostain and CTNNB1 mutation is controversial.

Moreno-Bueno et al. [50] and Hassanein et al. [53] reported similar results, with nuclear/cytoplasmic and cytoplasmic/membranous positivity in basaloid and transitional cells, respectively. Park et al. observed a predominance of membranous immunoreactivity in transitional cells, but not in basaloid cells, without evidence of nuclear positivity [51]. Xia et al. reported a correlation between nuclear *β*-catenin expression and CTNNB1 mutation since no mutations were detected in cases without *β*-catenin nuclear staining, suggesting that its nuclear localization could be considered a predictor of gene mutation [52]. Kim et al. observed nuclear and membranous strong positivity only in basaloid cells, with weak cytoplasmic expression [54].

These contrasting results may arise from technical differences, such as the stain method and/or anti-*β*-catenin antibodies.

Two of our cases with p.S33F CTNNB1 mutation showed cytoplasmic and equivocal nuclear immunostaining of *β*-catenin in basaloid cells. Interestingly, in the case of p.S45F mutation, a membranous staining pattern of *β*-catenin was observed in the same cells.

Nakamura and Fujiwara studied immunohistochemical localization of phosphorylated (pBC) and unphosphorylated (npBC) *β*-catenin [55]. They observed cytoplasmic, nuclear, and membranous expression of pBC in basaloid cells. Transitional cells showed membranous positivity for pBC and npBC. They proposed that npBC plays an important role in cell adhesion, and pBC is associated with apoptosis of basaloid cells, arguing that although BC is accumulated in the nucleus, post-translational modification or conformational changes may result in loss of or masked antigenicity and absence of nuclear immunoreactivity for npBC.

It is not possible to assess the status of the protein in our cases, whether phosphorylated or not, since we used a commercial antibody in our immunostaining, normally applied to a routine workflow for tumor diagnosis. This information could be interesting for further evaluation because *β*-catenin degradation depends upon its phosphorylation in the WNT pathway.

In the light of our observations, we speculate that another possible explanation for discrepancies of protein immunostain is that CTNNB1 mutations occurring in diverse and specific domains may confer different expression patterns of *β*-catenin, as detected by immunohistochemistry but lead to the same gain-of-function of the protein.

Our three cases of pilomatrixoma were diagnosed successfully by a combination of factors: awareness of the entity, usual clinical presentation, FNAB procedure, examination performed by the same cytopathologist, and maximum use of the obtained samples, through multiple preparation types, to facilitate the search for morphological findings, while still enabling additional studies.

Ieni et al. discussed the limitations of FNA in a series of 25 cases, pointing out that the most dangerous mistake is diagnosing a primary or metastatic malignant cutaneous tumor [43], stressing what was initially reported by Woyke et al. [7].

A review of the literature focusing on FNA diagnosis of pilomatrixoma revealed almost 200 reported cases. The extracted data confirmed previous observations. There is a wide range of ages, from 2 months to 76 years, with a predominance of young age, mainly the first 2 decades. Females were more commonly affected, approximately twice, and the head and neck/upper extremity locations were far more common than other sites (6 times). The size of the lesions varied broadly, from 0.5 to 9.0 cm in diameter.

Approximately one-third of the cases were misinterpreted, frequently as malignant (60%). Since PET/CT scan is now widely used in the workup of malignancy or suspicious lesions, and pilomatrixoma lesions may show intense abnormal FDG uptake, clinical correlation is essential because it is well established that false-positive results frequently occur secondary to nonmalignant processes that increase glucose uptakes, such as infection, inflammation, lymphoid follicular hyperplasia, and granulomatous diseases [4–6].

Five publications presented a more significant series, totaling 87 cases [20, 25, 27, 39, 43]. These authors described the diagnostic morphological findings that allowed a confident cytological diagnosis of pilomatrixoma, along with characteristic clinical presentation, including the presence of basaloid cells, shadow or ghost cells, acidophilic fibrillar substance, squamous cells, naked nuclei, calcium deposits, and inflammatory reaction with giant cells.

In the absence of all morphological findings or when one feature predominates, diagnosis can be very difficult [20, 27, 39]. For instance, when basaloid cells, giant cells, or ghost cells dominate, differential diagnosis may include other basaloid tumors, such as basal cell carcinoma or Merkel carcinoma, giant cell tumor, squamous cell carcinoma, or epidermal inclusion cyst, especially in cases with atypical clinical presentation or misinterpretation of clinical findings. The number of basaloid cells decreases, and that of ghost cells increases, with time, as previously observed [8]. Therefore, in older pilomatrixomas, tumor history and tumor size should be correlated with cytological composition. Morphometric analysis did not prove to be helpful [37].

The use of different cytological preparations, including conventional smears and cell blocks, can be very helpful [56]. The importance of examining Papanicolaou- and Diff-Quik-stained smears has been previously stressed, and the morphological findings are complementary and confirmatory [19, 39]. Liquid-based cytology is also valuable for conclusive diagnosis, especially when cellularity is scarce or discrete [48]. Cell blocks are relevant not only to screen for additional morphological findings, to better appreciate the uniformity of basaloid cells, ghost cells, and calcifications [27, 29], but also to perform complementary studies.

Only two patients underwent surgery, and the cytological diagnosis was confirmed. Although the remaining case presented typical cytomorphological and clinical findings, and clinical follow-up with observation was initially considered, we contacted the responsible physician to communicate our results and possible long-term risks, after successfully demonstrating *FGFR2* gene mutation.

Initiating *β*-catenin mutations may result in the development of pilomatrixomas in younger patients, which over time, with additional mutations, could progress into malignancy and formation of pilomatrical carcinoma, which is reported in rare cases [64, 65]. Pilomatrical carcinoma is an aggressive neoplasm; therefore, careful clinical evaluation is advisable. We did not identify TP53 and SMAD4 mutations in our cases, which have been associated with malignant neoplasms. Further, our panel did not include CDKN2A, ERBB4, and PTCH1, which have also been reported in these malignancies.

Immunohistochemistry can be particularly helpful in limited biopsy specimens, including cell blocks obtained by FNAB, especially in difficult cases; for example, *β*-catenin is expressed in pilomatrixoma, but not in basal cell carcinoma, squamous carcinoma, and small cell carcinoma and other entities with similar morphology [48]. Similar is the case with pilomatrical carcinoma, which shows diffuse BerEp4 and p53 positivity but lacks *β*-catenin expression [66].

In conclusion, pathology is continuously incorporating knowledge through molecular diagnostics. By combining traditional and new technologies, pathologists integrate the morphological, clinical, and molecular characteristics of the disease. In recent years, the morphomolecular approach has a central role in diagnosis and therapeutics. Minimally invasive procedures such as FNAB are increasingly used to obtain tissue samples to study. We were able to establish a preoperative diagnosis of pilomatrixoma by FNA, which represents an attractive alternative for punch or excisional biopsy by being simple, noninvasive, fast, and accurate in experienced hands. In addition to routine cytopathological evaluation, FNA specimens can be successfully used to detect the molecular and protein expression of CTNNB1 and *β*-catenin, respectively, which could be valuable for differentiation from other entities and proper management of the patient.

## Figures and Tables

**Table 1:** Age/sex (years; F = female), lesion size, immunohistochemistry for *β*-catenin, and mutational analysis of CTNNB1 are summarized for three cases of pilomatrixoma. The number of cells and percentage of tumor cells present on conventional smears used for molecular testing and the obtained DNA input are also reported.

Case ID	Age/sex	Lesion size (cm)	Immunohistochemistry for *β*-catenin	Number of cells	Tumor cells (%)	DNA input (ng)	*CTNNB1* mutation	Resection and histologic correlation	Other findings
1	6/F	1.0	Cytoplasmic and equivocal nuclear staining	15	60	100	c.98C>T, p.(Ser33Phe)	No	No
2	13/F	1.4	Cytoplasmic and equivocal nuclear staining	5	80	81.5	c.98C>T, p.(Ser33Phe)	Yes	No
3	38/F	1.4	Membranous staining	3	70	16.7	c.134C>T, p.(Ser45Phe)	Yes	FGFR2 p.S252L

## Supplementary Materials

Supplementary Materials Supplementary table 1: list of genes and respective covered exons used in the panel for solid tumors at the Molecular Pathology Laboratory of Hospital Sírio-Libanês.
