# Supplementary material for: Novel Mutations in Pilomatrixoma, CTNNB1 p.s45F, and FGFR2 p.s252L: A Report of Three Cases Diagnosed by Fine-Needle Aspiration Biopsy, with Review of the Literature
Source: Case Rep Genet. 2020 Aug 29;2020:8831006. doi: 10.1155/2020/8831006 (PMC7475758; doi:10.1155/2020/8831006)
Supplement: Supplementary Materials — Supplementary table 1: list of genes and respective covered exons used in the panel for solid tumors at the Molecular Pathology Laboratory of Hospital Sírio-Libanês. [file 8831006.f1.docx]

**SUPPLEMENTARY TABLE 1**

Supplementary table 1: List of genes and respective covered exons used in the panel for solid tumors at Molecular Pathology Laboratory of Hospital Sírio-Libanês.

| *AKT1* | exon 3 |
| --- | --- |
| *ALK* | exon 23 |
| *APC* | exon 15 |
| *BRAF* | exons 11 and 15 |
| *CDH1* | exons 8, 9 and 12 |
| *CTNNB1* | exon 2 |
| *EGFR* | exons 18 to 21 |
| *ERBB2* | exon 20 |
| *FBXW7* | exons 7 to 11 |
| *FGFR2* | exon 6 |
| *FOXL2* | exon 1 |
| *GNAQ* | exons 4 to 6 |
| *GNAS* | exons 6 and 8 |
| *KIT* | exons 9,11,13,17 and 18 |
| *KRAS* | exons 1, 2, 3, and 4 |
| *MAP2K1* | exon 2 |
| *MET* | exon 14 andintron 14-15 |
| *MSH6* | exon 5 |
| *NRAS* | exons 1, 2, 3, and 4 |
| *PDGFRA* | exons 12,14 and 18 |
| *PIK3CA* | exons 2,3,8,10 and 21 |
| *PTEN* | exons 1 to 7 and 9 |
| *SMAD4* | exons 8 and 11 |
| *SRC* | exon 10 |
| *STK11* |  |
| *TP53* |  |
